# Macrophages rescue cells from ferroptotic death

**DOI:** 10.1038/s41419-025-08277-6

**Published:** 2025-12-01

**Authors:** Ruth Hefetz, Sapir Lianski, Lucy Ghantous, Yael Volman, Yoel Sadovsky, Ofer Beharier, Jacob Rachmilewitz

**Affiliations:** 1 Goldyne Savad Institute of Gene Therapy, Hadassah Medical Center, Faculty of Medicine, Hebrew University of Jerusalem, Jerusalem, Israel; 2 Department of Obstetrics and Gynecology, Hadassah-Hebrew University Medical Center, Jerusalem, Israel; 3 Pediatrics, Division of Neonatal and Developmental Medicine, Stanford University, Stanford, CA USA

**Keywords:** Cell death, Innate immunity

## Abstract

Ferroptosis, a non-apoptotic form of cell death marked by iron-dependent lipid peroxidation, has a key role in organ injury, degenerative disease, and vulnerability of therapy-resistant cancers. Although substantial progress has been made in understanding the molecular processes relevant to ferroptosis, additional cell-extrinsic processes that determine cell sensitivity toward ferroptosis remain unknown. Here we demonstrate that macrophages co-cultured with ferroptotic cancer cells from various types effectively mitigate cell death induced by GPX4 inhibitors (RSL3 and ML162), GPX4 silencing via shRNA, or the Xc- system inhibitor IKE. Furthermore, macrophages effectively reduced lipid peroxidation in ferroptotic cells. Importantly, macrophage function relies on direct cell-to-cell contact and is affected by their differentiation. Specifically, polarization into M1 macrophages, but not M2, greatly hinders their protective capabilities. Interestingly, unlike apoptotic cells, ferroptotic cells retain elevated levels of the ‘don’t eat me’ signal, CD47, and conversely, fail to present the “eat me” signal phosphatidylserine (PS) on the outer layer of the plasma membrane, providing an opportunity for their rescue. Furthermore, in placental villi explants, macrophages protect trophoblasts from ferroptotic death. These results underscore the intricate interplay between ferroptotic cells and their microenvironment and provide compelling evidence of a yet-unrecognized anti-ferroptotic activity of macrophages as a cell-extrinsic mechanism that could be exploited by cancer cells to escape ferroptosis.

## Introduction

Ferroptosis is an iron-dependent cell death pathway, defined by excessive accumulation of lipid hydroperoxides that promotes membrane injury and cell death. Ferroptosis is a non-apoptotic mode of cell death that is different from necrosis, apoptosis, and autophagy in cell morphology and function [1–3].

Glutathione peroxidase 4 (GPX4) is a selenium-dependent glutathione peroxidase that catalyzes the reduction of peroxides at the expense of glutathione. Importantly, GPX4 directly reduces membrane-bound phospholipid hydroperoxides and thus protects against membrane damage [4–6]. Accordingly, GPX4 is also called phospholipid hydroperoxide glutathione peroxidase. Nuclear factor erythroid 2 (NFE2)-related factor 2 (NRF2) is a transcription factor predominantly affecting the expression of antioxidant genes and thus plays a significant role in the control of redox balance. Together, GPX4 and nuclear factor erythroid 2-related factor 2 function as negative regulators of ferroptosis by limiting ROS production. Accordingly, ferroptosis can be induced experimentally through the blockage of GPX4 by compounds such as erastin and Ras-selective lethal small molecule 3 (RSL3), resulting in a decrease in antioxidant capacity and accumulation of reactive oxygen species (ROS) in cells, ultimately leading to oxidative cell death [1–3].

Ferroptosis is related to the pathophysiology of many diseases, including cancer. Many of the cancers resistant to conventional treatment (i.e., glioblastoma) are highly sensitive to ferroptosis. It is proposed that malignant cells that have undergone metabolic reprogramming, which allow them to persistently survive chemotherapy, uniquely become sensitive to death via ferroptosis [7–12]. These observations mark ferroptosis activation as a promising novel anti-cancer tool and highlight the importance of understanding molecular and cellular mechanisms regulating cell ferroptosis. Although substantial progress has been made in understanding the molecular processes relevant to ferroptosis, additional cell-extrinsic and cell-intrinsic processes that determine cell sensitivity toward ferroptosis remain unknown.

Early research investigating the contribution of macrophages to tissue repair focused on their role as scavenger cells that phagocytize cellular debris, invading organisms, neutrophils, and other apoptotic cells after tissue injury. However, it is now clear that macrophages exhibit much more complex and unique roles in tissue homeostasis, repair, and cell fate [13]. For example, tumor-associated macrophages were found to play a crucial key role in cancer biology, facilitating cancer growth and metastatic spread [14]. Moreover, we have previously demonstrated that cells injured by oxidized stress attract macrophages via the release of lipid metabolites [15]. These recruited macrophages facilitate DNA damage repair in the neighboring cell, leading to higher levels of double-strand breaks rejoining and improving cell survival [16, 17]. Others have shown that ferroptotic cells release a variety of damage-associated molecular patterns (DAMPs) and lipid metabolites that can mediate the recruitment of macrophages to the site of injury [18, 19]. However, the role of macrophages in this process is still not clear.

## Results

**Fig. 1:**
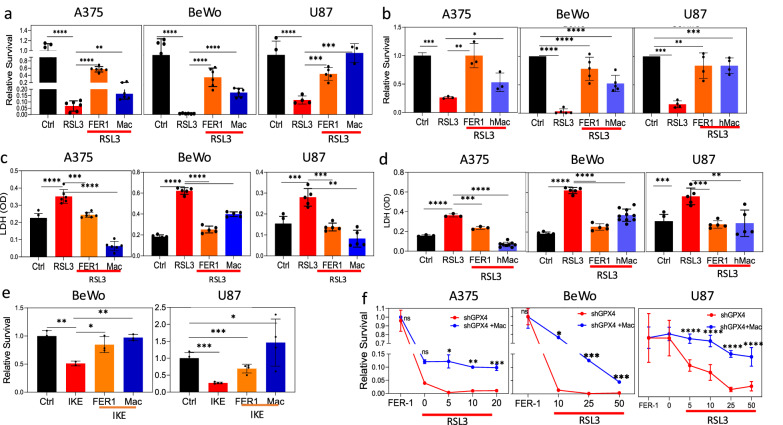
Macrophages rescue cells from ferroptotic death. **a** The indicated cell types were treated with the RSL3 for 1 h and then were incubated for 24 h with and without Far-red-labeled THP-1-derived macrophages. Cell survival was determined by flow cytometry after gating out macrophages. Graphs summarize an average of 6, 5, and 4 independent experiments, respectively. Rescue by Fer1 was used as a control. **b** Cells were treated as in (**a**) and were co-cultured with monocyte-derived instead of THP-1-derived macrophages. **c**, **d** Cell death in (**a**, **b**) was determined by LDH release. **e** Cells were treated with IKE for 1 h and then incubated for 24 h with and without Far-red-labeled THP-1-derived macrophages. Cell survival was determined by flow cytometry after gating out macrophages. **f** Macrophages rescue cells induced to ferroptosis by GPX4 silencing and Fer1 withdrawal with or without low doses of RSL3. Graphs in (**b**–**f**) show an average (±STD) of at least four replicates in each group. Representative of at least three independent experiments is shown. ns: non-significant; *p < 0.05; **p < 0.01; ***p < 0.001; ****p < 0.0001.

We postulated that macrophages may interact with cells undergoing ferroptosis and possibly attenuate ferroptotic death. To test this notion, we examined whether co-culturing ferroptotic cells with macrophages improves their survival. We employed three distinct cell lines—A375 (melanoma), BeWo (choriocarcinoma), and U87MG (glioblastoma)—chosen for their diverse tissue origins and demonstrated sensitivity to ferroptotic cell death. Cells were pretreated with various doses of RSL3 (for 1 h), washed, and then incubated alone or co-cultured with macrophages. The pretreatment strategy was based on the need to target cancer cells and avoid modulation of the macrophages by the RSL3. THP-1-derived macrophages (Mac) were labeled with CellTrace™ Far Red to distinguish them from the ferroptotic cells during flow cytometric analysis (Fig. S1). Ferrostatin-1 (Fer1), a potent and selective ferroptosis inhibitor [1, 20], was used as a control, confirming that cell death indeed results from ferroptosis. After 24 h in culture, the number of surviving cells was determined using flow cytometric analysis after gating out CellTrace-labeled macrophages. THP-1-derived macrophages rescue the various types of cells from ferroptotic death induced by a wide range of RSL3 doses (Fig. 1a and Fig. S2a). Interestingly, the three cell types differ in their sensitivity to RSL3-induced ferroptosis as well as in the efficiency of rescue by macrophages. Similar results were obtained when monocyte-derived macrophages were used instead of THP-1-derived macrophages (Fig. 1b). Lactate dehydrogenase (LDH), a cytoplasmic enzyme that is released into the cell culture supernatant when the plasma membrane is damaged, was also tested. Treatment with RSL3 resulted in an elevated release of LDH into the media, indicating increased cell death. Notably, the presence of either Fer1, THP-1-derived macrophages (Fig. 1c), or monocyte-derived macrophages (Fig. 1d) resulted in a reduced LDH release. Similar results were achieved by utilizing an additional GPX4 inhibitor, ML162, instead of RSL3 to induce ferroptosis in the cells (Fig. S2b, c). Ferroptosis can also be induced by inhibition of the cystine-glutamate antiporter, system xc-. We further assessed the effect of macrophages on ferroptosis induced by targeting system xc-, using Imidazole ketone erastin (IKE), a potent metabolically stable inhibitor of system xc-. THP-1-derived macrophages rescued both BeWo and U87MG cells from ferroptotic death induced by IKE, as measured by the number of surviving cells (Fig. 1e). Of note, A375 cells are not susceptible to ferroptotic death induced by IKE. Alternatively, we silenced GPX4 expression in A375 and BeWo cells using lentivirus-driven shRNA and induced ferroptosis in these cells by Fer1 withdrawal [21] in the absence or presence of low RSL3 doses. THP-1-derived macrophages similarly rescued these cells from ferroptotic death (Fig. 1f).

We next investigated whether other forms of cell death were involved. We co-treated cells with RSL3 and either the apoptosis inhibitor Z-VAD-FMK [22], the autophagy inhibitor bafilomycin A1 (Baf-A1) [23], or the ferroptosis inhibitor Fer1 (Fig. S3). While a modest improvement in cell viability was observed with RSL3 and Z-VAD-FMK co-treatment, the rescue effect was substantially greater when cells were treated with ferrostatin-1. In contrast, Baf-A1 failed to rescue cells from RSL3-induced cell death.

**Fig. 2:**
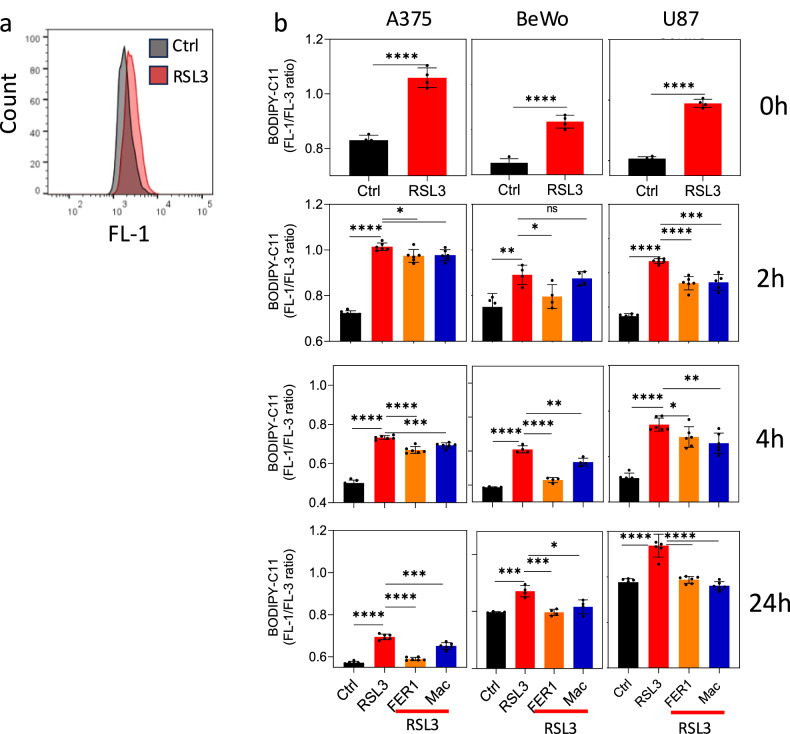
Macrophages reduce lipid peroxide in cells undergoing ferroptosis. BODIPY-labeled cells were treated as in Fig. 1a and then analyzed by flow cytometry. **a** Representative flow cytometric histogram of control BeWo cells and cells treated with RSL3. **b** Graphs illustrate the quantitative analysis of C11-BODIPY signal intensity at the indicated time points during ferroptosis based on flow cytometry experiments. Time 0 refers to the cells immediately after RSL3 pretreatment and before the addition of either Fer1 or macrophages to the culture. Representative of three independent experiments is shown.

Ferroptosis is associated with the accumulation of lipid peroxides [24, 25]. We therefore measured the level of lipid peroxidation by staining cells with the lipid dye BODIPY-C11. The level of lipid peroxidation increased in RSL3-treated cells immediately after the end of the pretreatment (time 0). While the level of lipid peroxidation was increased in RSL3-treated cells, with time it was significantly reduced in the presence of either Fer1 or THP-1-derived macrophages (Fig. 2). Given that oxidative damage appears prior to adding macrophages to the co-culture, these findings suggest that macrophages assist ferroptotic cells to either repair existing damage or prevent additional accumulation of lipid peroxide.

**Fig. 3:**
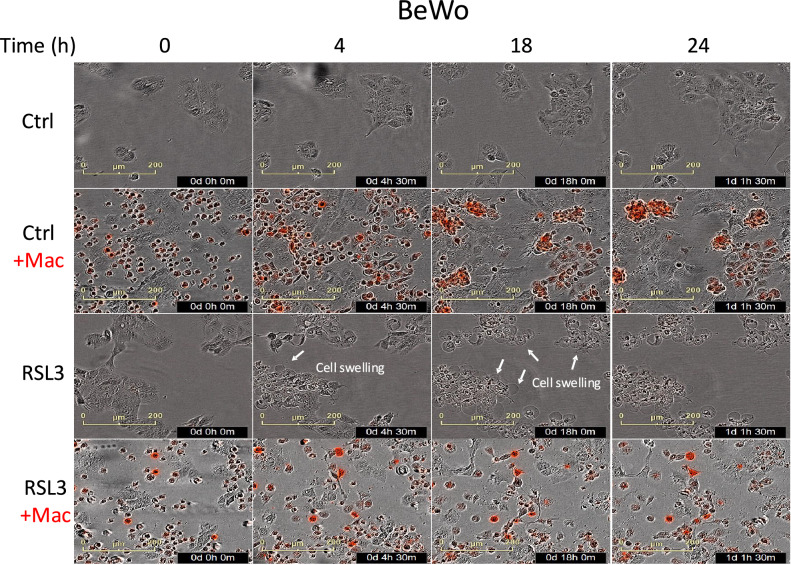
Imaging of macrophages adhering to and rescuing cells undergoing ferroptosis. BeWo cells were either left untreated or treated with RSL3 for 1 h and then washed. CellTrace-Red-labeled macrophages were then added (designated as time 0), and the co-cultures were incubated for 24 h. Live-cell imaging was performed with IncuCyte.

We further utilized microscopy in conjunction with live-cell imaging (Fig. 3, Fig. S4, and Supplementary Movies 1–7). Ferroptosis is characterized by a significant alteration in the plasma membrane, marked by blebbing and vesiculation, appearing as early as 4 h after ferroptosis induction, as reported previously [26]. Macrophages clearly adhered in clusters on both control and ferroptotic cells. Notably, in the presence of macrophages, cells’ blebbing was reduced, and more cells survived, providing further support for the role of macrophages in guarding cells against ferroptosis. Collectively, our data show that macrophages rescue cells from ferroptotic stress.

**Fig. 4:**
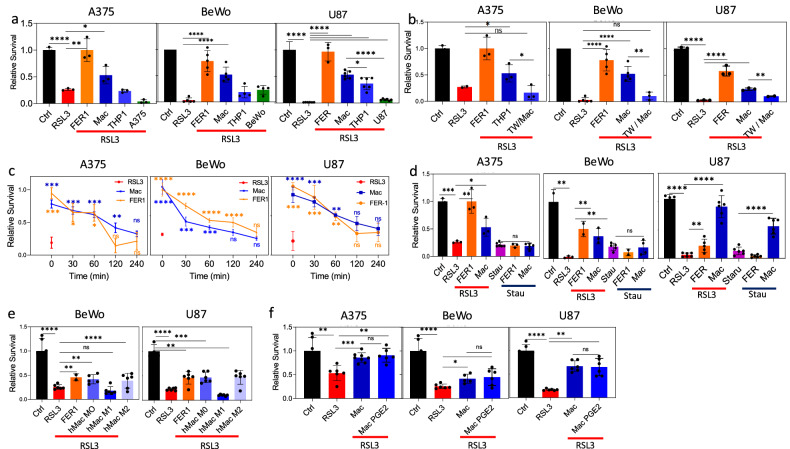
Characterization of macrophage-mediated rescue of ferroptotic cells. **a** Cells were treated as in Fig. 1a and then co-cultured with either THP-1-derived macrophages, undifferentiated THP-1, or with the same type of cells. **b** Ferroptotic cells and macrophages were co-cultured together or on opposite sides of a transwell membrane for 24 h, and cell survival was determined as above. **c** Cells were treated with RSL3 for 1 h as described above, then washed, and Fer1 and macrophages were added at various time points after ferroptosis induction to assess the time window during which macrophages can still rescue ferroptotic cells. **d** Cells were treated with either RSL3 or staurosporine, then washed, after which Fer1 or macrophages were added. Both Fer1 and macrophages were unable to rescue apoptotic cells. **e** Monocyte-derived macrophages were polarized to M1 using IFN-γ and LPS, to M2 using IL-4 and IL-13, or left untreated (M0), and then added to cell cultures that had been pretreated with RSL3 as described above. **f** M0 monocyte-derived macrophages were treated with PGE2 for 24 h prior to adding them to ferroptotic cells. Ferroptotic cell survival was tested after 24 h of co-culture, as above. Representative of at least three independent experiments is shown. ns non-significant; *p < 0.05; **p < 0.01; ***p < 0.001; ****p < 0.0001.

**Fig. 5:**
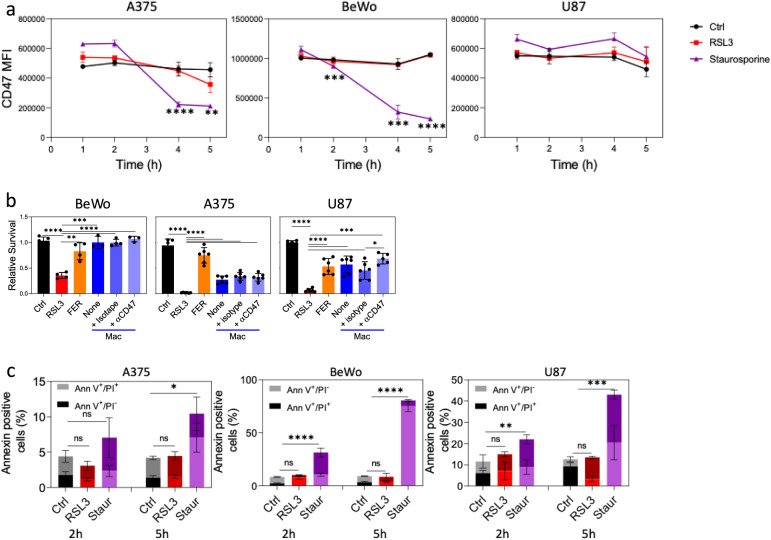
Expression of ‘don’t eat me’ and ‘eat me’ signals on ferroptotic cells. **a** CD47 expression before (control) and 2 h and 5 h after ferroptosis (RSL3) or apoptosis (Staurosporine) induction in BeWo and A375 cells. Graphs show an average (± STD) CD47 median fluorescence intensity (MFI) of 4 replicates in each group. **b** The indicated cell types were treated with RSL3 (500 nM) for 1 h, followed by preincubation with either blocking anti-human CD47 antibodies (5 µg/ml) or IgG1κ isotype control antibodies (5 µg/ml) for 15 min. Subsequently, Far-red-labeled THP-1-derived macrophages were added. Cell survival was assessed by flow cytometry as described above. Similar results were obtained when 10 and 20 µg/ml blocking antibody was used. Graphs display an average (±STD) of 6 replicates in each group. **c** Cells were treated as in (**a**) and were then stained with annexin V and PI. The percentage of annexin-positive cells is shown. Graphs display an average (±STD) of 4 replicates in each group. Representative of at least three independent experiments is shown. ns non-significant; *p < 0.05; **p < 0.01; ***p < 0.001; ****p < 0.0001.

To determine whether this effect is specific to macrophages, immediately after ferroptosis induction as above, ferroptotic cells were co-cultured with CellTrace-labeled THP-1 cells that were not treated with PMA and did not differentiate into macrophages, or with CellTrace-labeled cells of the same type (i.e., BeWo, A375, or U87MG cells) that were not treated with RSL3. Only PMA-treated THP-1 cells that differentiated to macrophages were able to significantly rescue cells from ferroptotic death (Fig. 4a). By altering the number of macrophages added to the co-culture, we determined that the optimal cell:macrophage ratio required for ferroptotic cell rescue is around 1:3 (Fig. S5). Importantly, cell rescue requires cell:macrophage contact, given that the effect is abrogated when macrophages and ferroptotic cells are physically separated on opposite sides of a transwell membrane (Fig. 4b). This latter finding is supported by the microscopy observations shown in Fig. 3, which demonstrate close adherence of macrophages to the ferroptotic cells. Cell rescue by macrophages was limited to the first 2–3 h after ferroptosis induction, as macrophages added at later time points were unable to rescue ferroptotic cells (Fig. 4c), probably reflecting irreparable changes within the ferroptotic cells. Importantly, both Fer1 and macrophages failed to rescue cells induced to apoptosis by staurosporine (Fig. 4d), with the exception of staurosporine-treated U87MG cells, which were rescued by macrophages (see also Fig. 5a). To assess the impact of macrophage polarization on their ability to rescue ferroptotic cells, we repeated the experiments described above using M0 monocyte-derived macrophages that were polarized toward M1 or M2. The polarization was achieved using IFNγ and LPS for M1, or IL-4 and IL-13 for M2 (Fig. S6), as outlined in the “Materials and methods” section. We then added these macrophages to ferroptotic cells and compared the number of surviving cells. While there was no significant difference in the rescue efficiency between M0 and M2 macrophages, M1 macrophages exhibited significantly lower rescue efficiency (Fig. 4e). A recent study reported that within the tumor microenvironment, neutrophils undergo spontaneous ferroptosis, releasing prostaglandin E2 (PGE2), which in turn regulates the activity of CD8^+^ T cells and tumor-associated macrophages [27]. Additionally, PGE2 has been implicated in the polarization of M2 macrophages [28]. Therefore, we investigated whether PGE2 influences the efficiency of rescue by macrophages. To this end, macrophages were treated with PGE2 for 24 h before being added to the co-culture with RSL3-treated cells. The PGE2 treatment did not significantly impact the ability of macrophages to rescue ferroptotic cells (Fig. 4f).

CD47 is a cell surface protein that acts as a “don’t eat me” signal that protects cells from phagocytosis by binding and activating its receptor SIPRα on macrophages. Surface CD47 level is reduced when apoptosis occurs, enabling phagocytosis of apoptotic cells. Intriguingly, our results demonstrate that, unlike apoptotic cells that downregulate the expression of CD47 early after apoptosis induction, ferroptotic cells maintain high levels of CD47 (Fig. 5a and Fig. S7). As anticipated, U87MG cells maintain CD47 expression following RSL3-induced ferroptosis; however, they also unexpectedly retain CD47 levels during apoptosis. This may account for the macrophage-mediated rescue of staurosporine-treated apoptotic U87MG cells, as shown in Fig. 4d. Given that ferroptotic cells maintain CD47 expression level, we tested the role of CD47 in macrophage-mediated rescue. Surprisingly, adding the neutralizing anti-CD47 antibody (clone B6.H12), which blocks CD47–SIRP-α interaction and promotes phagocytosis [29], had no effect on macrophage-mediated rescue in the co-culture (Fig. 5b), suggesting that in the context of ferroptosis, CD47 might not be crucial for this rescue process. This may suggest that additional pathways or signals are necessary to prevent macrophage phagocytosis and provide time for cell rescue. Since phagocytosis is regulated by the balance of ‘don’t eat me’ and ‘eat me’ signals on the surface of host cells [30]. Therefore, we also examined the exposure of the ‘eat me’ signal, the anionic phospholipid phosphatidylserine (PS), on ferroptotic cells by labeling them with the PS-binding protein annexin V. For that end, we induced both apoptotic and ferroptotic cell death as described earlier and then stained the cells with FITC-labeled annexin V and propidium iodide (PI) (Fig. 5c). In contrast to apoptotic cells, the ‘eat me’ signal does not appear on the surface of ferroptotic cells during the initial 5 h, when the ferroptotic process is still reversible (Fig. 4c). Given that CD47 downregulation and PS upregulation are linked to enhanced phagocytosis of dying cells by macrophages, it is possible that ferroptosis suppress phagocytosis thus enabling ferroptotic cells overcome their stressful conditions.

**Fig. 6:**
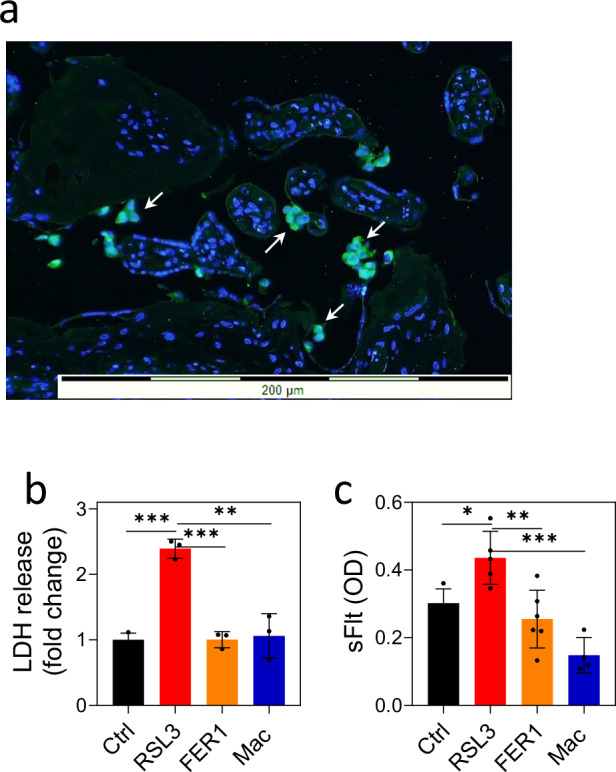
Macrophages adhere to placental explants and protect against ferroptotic injury and dysfunction. **a** Placental villi explants were treated ex vivo with RSL3 for 1 h and then incubated with GFP-expressing macrophages. After 2 h, the placental explants were collected, extensively washed, and fixed with paraffin. Tissue sections were stained using anti-GFP antibodies. The image illustrates macrophages adhering to the placental tissue (arrows). Placental villi explants were treated ex vivo as in (**a**) and then incubated without and with either Fer1 or macrophages. After 24 h, conditioned media were collected, and the level of LDH (**b**) and sFLIT (**c**) was determined. The amount of LDH and sFLIT in each well was normalized to the dry weight of the tissue. Graphs present the fold change of LDH release in each sample as compared to control untreated tissues, denoted as 1. The graph represents an average of three independent experiments and is presented as a fold change of LDH release in each sample as compared to control untreated tissues, denoted as 1. sFLIT secretion was determined by ELISA. Similar results were obtained in three independent experiments. *p < 0.05; **p < 0.01; ***p < 0.001.

To test whether macrophages rescue ferroptotic cells ex vivo, placental villi explants were incubated with RSL3 in the absence or presence of THP-1-derived macrophages. Approximately two hours later, macrophages firmly adhered to the tissue explants (Fig. 6a). After 24 h, the release of LDH was tested and was normalized to the explant protein content in each well. The level of LDH was significantly increased in explants treated with RSL3 as compared to control untreated explant villi, and this increase was reduced when villi explants were incubated with either Fer1 or macrophages (Fig. 6b). Soluble fms-like tyrosine kinase-1 (sFlt-1) is released in excess from the placenta in response to ischemic and hypoxic stress conditions [31, 32]. Therefore, supernatant sFlt-1 concentration was measured by ELISA. Importantly, the level of sFlt-1 increased upon RSL3 treatment but not in the presence of macrophages or Fer1, used as a control (Fig. 6c), suggesting macrophages protect placental tissue from ferroptotic injury.

## Discussion

Ferroptosis is an intracellular iron-dependent form of cell death that is distinct from apoptosis, necrosis, and autophagy. Extensive studies suggest that ferroptosis plays a pivotal role in tumor suppression. Specifically, a growing body of evidence has demonstrated that therapy-resistant cancer cells derived from a wide range of cancers and drug treatments, or have a high propensity to metastasize, exhibit increased sensitivity to ferroptosis [7, 9, 10]. Therefore, the exploration of ferroptosis and its regulatory mechanisms represents a cutting-edge and promising approach for cancer treatment. However, the precise mechanisms governing the dynamic regulation of ferroptosis susceptibility remain poorly elucidated [33]. Cell susceptibility to ferroptosis can be determined by either pre-existing cell-intrinsic characteristics across various cell lineages or by non-cell-autonomous external cues that extend beyond the cells themselves, mediated by components of the microenvironment, including immune cells [34]. However, most of the studies did not address the involvement of the tumor microenvironment or the immune system in regulating the susceptibility of cancer cells to ferroptosis.

Macrophages are a ubiquitous cellular component present in all tissues of the body, performing diverse functions to maintain homeostasis, and are an essential part of the tumor microenvironment. Here, we demonstrated that macrophages interact with various types of both normal and cancer cells undergoing ferroptosis, reduce lipid peroxidation, and make them less susceptible to death. Macrophages also decreased the membrane swelling characteristic of cells undergoing ferroptosis. While the exact molecular mechanism of macrophage rescue is still unclear, it is evident that this effect was specific to macrophages, was dependent on cell contact, and was restricted to the first 3–4 h after ferroptosis induction. This timeframe appears to be when the ferroptotic process is reversible, allowing for cell rescue.

This protective activity did not differ significantly between M0 and M2 macrophages, but it was notably reduced when macrophages differentiated into the M1 phenotype. An earlier study revealed that M1 macrophages, which express inducible nitric oxide synthase (iNOS), exhibit reduced susceptibility to ferroptotic cell death compared to iNOS-lacking M2 cells. Additionally, either Nitric Oxide (NO) donors or NO released from M1 macrophages were found to hinder ferroptotic death in nearby cells [35]. Conversely, a recent study revealed that ferroptosis induced by pharmaceutical inhibition of GPX4 increased when breast cancer cells were co-cultured with RAW 264.7 macrophages [36].

The difference between these studies and our investigation lies in the experimental procedure. While we utilized a pretreatment method to selectively target cancer cells without affecting macrophages, in the former studies, macrophages were also exposed to RSL3, potentially resulting in ferroptotic damage that may hinder their ability to assist neighboring cells. Our approach is based on the premise that ferroptosis is driven by the intracellular accumulation of endogenous lipid peroxides within organelles, rather than by external oxidative stress affecting all neighboring cells like macrophages in the tumor microenvironment. This concept is further supported by the observation that susceptibility to ferroptosis differs between cell types [37–40] and is primarily determined by intrinsic factors, such as metabolic state and redox homeostasis. Cells characterized by elevated levels of reactive oxygen species (ROS), impaired antioxidant defenses, and membranes enriched with polyunsaturated fatty acids (PUFAs) are especially prone to undergo ferroptosis. Consequently, tumor cells, owing to their distinct metabolic and redox characteristics, tend to be more prone to ferroptosis, whereas macrophages, although exposed to the same microenvironment, may remain unaffected. In this scenario, the exceptional case is represented by M1 cells, which exhibit resistance to ferroptosis and can protect neighboring cells from ferroptosis through the release of NO [35]. Our data present an additional mechanism that is distinct but not mutually exclusive from the one mentioned above and is based on cell-to-cell contact.

A recent study found that neutrophils are particularly susceptible to ferroptosis and undergo this process spontaneously within the tumor microenvironment. In turn, ferroptotic neutrophils release the immunosuppressive molecule PGE2, which promotes suppressive activity in macrophages, inhibiting T cell function and facilitating tumor growth [27]. We proposed that in this context, PGE2 could boost the protective activity of tumor macrophages. To explore this, we examined whether PGE2 influences the efficacy of macrophage rescue. However, PGE2 treatment did not significantly impact their protective activity.

One of macrophages' primary functions is to act as scavengers, ingesting and breaking down foreign substances, as well as damaged or dying cells. Indeed, apoptotic cells have decreased levels of the ‘don’t eat me signal’, CD47, which allows their clearance by macrophages. Surprisingly, we found that ferroptotic cells retain normal surface expression levels, which may help them evade phagocytosis by macrophages and allow time for recovery. Notably, under our experimental conditions, U87MG cells undergoing apoptosis also retained CD47 expression and were similarly rescued by macrophages. However, our data also suggest that CD47 alone is not sufficient, since macrophages are still able to rescue cells from ferroptotic death even when CD47 on the target cells is blocked. This suggests that there could be other ‘don’t eat me’ signals like PD-L1, or beta-2 microglobulin (B2M) [41], or other mechanisms enabling ferroptotic cells to avoid phagocytosis. Indeed, we show that ferroptotic cells do not present the ‘eat me’ signal phosphatidylserine (PS), on the outer layer of the plasma membrane for a duration (of 3–4 h after ferroptosis induction) that is sufficient to enable macrophages to rescue them from damage. These results resemble those reported in a previous study, which show that in Jurkat cells, surface PS is visible on ferroptotic cells only 5 h after RSL3 treatment, whereas it appears on cells induced to undergo apoptosis by anti-Fas antibodies already at 3 h [42].

In conclusion, the role of macrophages in the tumor microenvironment, cancer progression, and treatment resistance is gaining increasing recognition. Understanding the metabolic and molecular changes, as well as the cellular interactions that regulate antitumor immunity, paves the way for the development of innovative cancer cell therapies and immunotherapies.

Our findings suggest that macrophages may impede efforts to combat cancer using ferroptosis inducers. This highlights the need for current initiatives aimed at translating pharmacological induction of ferroptosis in cancer cells into clinical practice to consider the involvement of immune cells, particularly tumor-associated or recruited macrophages.

## Materials and methods

### Cell culture

The human Glioblastoma cell line (U87MG; ATCC; HTB-14) and the human Malignant Melanoma cell line (A375; ATCC; CRL-1619) were maintained in a high-glucose DMEM medium (Life Technologies, Grand Island, NY). The human placenta cell line (BeWo; ATCC; CCL-98) was maintained in ATCC-formulated F-12K Medium (ATCC, United States). The human monocyte (THP-1; ATCC; TIB-202) was maintained in RPMI 1640 Medium (Life Technologies, Grand Island, NY). All media were supplemented with 10% heat-inactivated fetal calf serum, 1% sodium pyruvate, and 1% penicillin-streptomycin (Biological Industries, Beit-Haemek, Israel) at 37 °C and 5% CO_2_.

### Macrophage preparation

For the preparation of THP-1-derived macrophages, 10^7^ THP-1 cells were labeled with cell trace (CellTrace™ Far Red Cell Proliferation Kit, C34564, Thermo Fisher Scientific Inc), and plated in a 100 mm plate in the presence of 5 ng/ml phorbol 12-myristate 13-acetate (PMA, P8139, MERCK). After 48 h, the cells were trypsinized, counted, and replated as indicated.

Preparation of monocyte-derived macrophages - Ethical clearance for the use of human subjects was obtained from the Hadassah-Hebrew University committee. Peripheral blood was obtained from buffy coats of healthy donors, and peripheral blood mononuclear cells (PBMCs) were isolated by density gradient centrifugation using Histopaque (1.077 g ml^−1^; Sigma-Aldrich) as previously described [43]. 10^7^ PBMCs were then allowed to adhere in a 100 mm plate for 50 min in serum-free medium. The non-adherent cells were then removed by repeated washing, and the remaining cell population, enriched with monocytes, was then cultured in RPMI 1640 medium supplemented with M-CSF (50 ng/ml; PeproTech, Rocky Hill, NJ) and G-CSF (50 ng/ml; PeproTech) for 7 days. The medium was replaced with fresh medium every 2 days. Macrophages were then gently scraped, labeled with cell trace (CellTrace™ Far Red), and replated as indicated.

For macrophage polarization, cells were either left untreated (M0), treated with Interferon-γ (IFNγ; 50 ng/ml; PeproTech) and LPS (20 ng/ml; Sigma-Aldrich) for M1 polarization or with IL-4 (20 ng/ml; PeproTech) and IL-13 (20 ng/ml; PeproTech) for M2 polarization, for 48 h. Subsequently, the cells were characterized via flow cytometric analysis.

### GPX4 shRNA

Silencing GPX4 was performed using MISSION shRNA plasmids (Sigma-Aldrich). Lentiviral vectors (pLKO.1) encoding either a nontargeting shRNA (SHC016, Sigma-Aldrich) or shRNA directed against human GPX4 (TRCN0000046251, designated shGPX4, Sigma-Aldrich) were co-transfected with pMD2.G and psPAX2 plasmids (Addgene 12259, 12260, respectively) into the HEK293T packaging cell line, using TransIT-Lenti transfection reagent (Mirus). After 48–72 h, viral particles were harvested from the culture supernatant by filtering through a 0.45-μm syringe filter and concentrated by centrifugation (18,000 rpm for 2 h), aliquoted, and stored at −80 °C. Viral particles, harboring either nontargeting control or GPX4-directed shRNA, were used to transduce BeWo cells following polybrene (8 μg/mL, Sigma-Aldrich) for 1 h. After 48 h, transduced cells were selected with 2 μg/mL puromycin (Sigma-Aldrich). Ferrostatin-1 (0.5 μM; Fer1; HY-100579, MedChemExpress, USA) was added to inhibit spontaneous ferroptosis.

### Ferroptosis induction

For ferroptosis induction, 55 × 10^3^ BeWo and A375 and 20 × 10^3^ U87MG cells were plated in a 24-well dish, and 24 h later were pretreated with 500 nM either RSL3 (HY-100218A, MCE, USA), ML162 (SML2561, Merck), IKE (1801530-11-9, Cyman, USA), or were mock treated with vehicle only in media containing 1% FCS. After 1 h, cells were washed with fresh media. In certain experiments, apoptosis was induced using 400 nM Staurosporine (AM-2882, MCE). Only at that stage, either Ferrostatin-1 (500 nM; HY-100579, MCE) or Cell-Trace labeled macrophages (150 × 10^3^) were added to the culture, and this time point is hence denoted as time 0. The cells were then incubated for the indicated time points, after which they were collected, and their relative numbers were measured using a CytoFLEX Flow Cytometer (Beckman Coulter, Indianapolis, USA) with time-based acquisition settings. The number of surviving cells was determined after gating for PI^-^ live cells and gating out CellTrace-labeled macrophages, using the CytExpert software as depicted in Supplementary Fig. 1a. In certain experiments, macrophages were placed in the top chamber of Transwell membranes (Corning; 0.4 mm pore size) of lower wells containing the various cell types. Additionally, in several experiments, cells were pre-incubated for 15 min with either anti-human CD47 antibodies (5–20 µg/ml; Clone B6.H12; Cat. No. BE0019-1, Bio X Cell) or isotype control antibodies (5–20 µg/ml; mouse IgG1κ; Cat. No. 400102, BioLegend), after which macrophages were added into the co-culture. In an additional set of experiments, cells were co-treated with RSL3 and either Z-VAD-FMK (20 µM; Cat. No. 16658B, MCE) or bafilomycin A1 (Baf-A1; 100 nM; Cat. No. 11038, Cyman). Cell death was further quantified by measuring released lactate dehydrogenase (LDH) activity in the media using the Cytotoxicity Detection Kit (LDH) according to the manufacturer’s instructions (Roche Diagnostics).

### Flow cytometry

Cells were trypsinized and stained with APC-conjugated anti-human CD47 monoclonal antibody (clone CC2C6; Cat No. 323102; Biolegend, San Diego, CA). For macrophage characterization, cells were immunostained with APC-conjugated anti-human CD163 mAb (clone GHI/61; Cat No. 333610; Biolegend), PE-conjugated anti-human CD86 mAb (clone BU63; Cat No. 374206; Biolegend), and FITC-conjugated anti-human CD11c mAb (clone ICRF44; Cat No. 301330; Biolegend). Cells were incubated for 30 min on ice, followed by two washes with PBS. Annexin V staining was performed using the eBioscience™ Annexin V Apoptosis Detection Kit FITC (88-8005-74) according to the manufacturer’s instructions. Flow cytometric analysis was conducted on a CytoFLEX Flow Cytometer (Beckman Coulter, Indianapolis, USA) using CytExpert software.

### Lipid peroxidation assay

Lipid peroxidation of cells was assessed using the fluorescent probe BODIPY (581/591) C11 (Invitrogen, D3861). Briefly, following trypsinization, the cells were washed twice using PBS ×1. The cell pellet was then resuspended in 1 mL of PBS ×1 containing a final concentration of 1 μM of BODIPY (581/591) C11 and incubated for 30 min at 37 °C. Afterward, 10 mL of media was added and the cells were incubated for an additional 10 min. Then, the cells were centrifuged at 1500 rpm for 5 min, resuspended in medium, and cells were plated and treated as described above. At the indicated time points, cells were trypsinized and analyzed by flow cytometry.

### Placental explants

#### Ex vivo studies

This study was approved by The Hadassah Hospital Medical Center Institutional Review Board, according to the principles of the Helsinki Declaration (0648-21-HMO). All patients gave written informed consent for placental biopsy collection. Biopsies (2–3 cm) were taken within 30 min post-delivery from a mid-region between cord insertion and placental margin. These biopsies served for placental explant preparation or snap freezing in liquid nitrogen for subsequent phospholipid analysis.

The biopsies served for placental explant preparation were thoroughly washed in ice-cold PBS before being dissected into small fragments of 1–2 mm, and then four pieces were transferred to each well in a 24-well plate. The placental explants were cultured in DMEM ×1 supplemented with 10% FBS and 1% antibiotic-antimycotic and maintained at 5% CO_2_ and 8% O_2_ (mimicking the oxygenation state after 14 weeks of gestation) for the first 72 h, with the medium being changed every 24 h.

On the fourth day of the experiment, the explants were washed once in PBS ×1 and transferred to an F-12K medium containing 1% FBS. To induce ferroptosis, RSL3 was added for 1 h. Then the media was aspirated and was replaced with fresh media, or media supplemented with Fer1 or with THP-1-derived macrophages. After an additional 24 h, the supernatants were collected and frozen (−80 °C) for future analyses.

### Immunofluorescence (IF) staining

IF analysis of placental explant tissues was performed as described previously [16, 17]. Briefly, Paraffin-embedded placental tissue sections were incubated with goat anti-GFP mAbs (ab6673; Abcam, Cambridge, UK), followed by a FITC-labeled anti-goat secondary antibody (705-545-147, Jackson Immuno Research, West Grove, PA). An Olympus BX61 microscope (Olympus) was used for image acquisition.

### Cell death assay

Cell death was measured using a colorimetric assay to quantify lactate dehydrogenase (LDH) activity released from the cytosol of damaged cells (Roche, 11644793001). The assay was performed according to the manufacturer’s instructions. Sample OD was measured at 540 nm using the BIOTEK ELx808 Microplate Reader.

### ELISA assays

Cell culture supernatants were collected as described above to analyze sFlt-1 release. An enzyme-linked immunosorbent assay was performed, following the manufacturer’s instructions (R&D Systems, DY321B). sFlt-1 release was matched and normalized per placenta protein content in each well.

### Statistical analysis

All data were subjected to statistical analysis using the Excel software package (Microsoft) or GraphPad Prism 10 (GraphPad Software Inc., La Jolla, CA, USA). A two-tailed unpaired Student’s t-test was used to determine the difference between the groups. Data are given as mean ± SD and are shown as error bars for all experiments. Differences were considered significant at P < 0.05; *P < 0.05, **P < 0.01, ***P < 0.001, ****P < 0.0001.

## Supplementary information

Supplementary data

Supplementary Movie 1_BeWo RSL3

Supplementary Movie 2_BeWo RSL3_MAC

Supplementary Movie 3_BeWo Control

Supplementary Movie 4_BeWo Control +Mac

Supplementary Movie 5_BeWo RSL3.

Supplementary Movie 6_BeWo RSL3 +Fer1

Supplementary Movie 7_BeWo RSL3 +Mac

## Data Availability

The data that support the findings of this study are not openly available and are available from the corresponding author upon reasonable request.

## References

[1] Dixon SJ, Lemberg KM, Lamprecht MR, Skouta R, Zaitsev EM, Gleason CE, et al. Ferroptosis: an iron-dependent form of nonapoptotic cell death. Cell. 2012;149:1060–72. 22632970 10.1016/j.cell.2012.03.042 PMC3367386

[2] Li J, Cao F, Yin HL, Huang ZJ, Lin ZT, Mao N, et al. Ferroptosis: past, present and future. Cell Death Dis. 2020;11:88. 32015325 10.1038/s41419-020-2298-2 PMC6997353

[3] Xie Y, Hou W, Song X, Yu Y, Huang J, Sun X, et al. Ferroptosis: process and function. Cell Death Differ. 2016;23:369–79. 26794443 10.1038/cdd.2015.158 PMC5072448

[4] Ursini F, Bindoli A. The role of selenium peroxidases in the protection against oxidative damage of membranes. Chem Phys Lipids. 1987;44:255–76. 3311419 10.1016/0009-3084(87)90053-3

[5] Forcina GC, Dixon SJ. GPX4 at the crossroads of lipid homeostasis and ferroptosis. Proteomics. 2019;19:e1800311. 30888116 10.1002/pmic.201800311

[6] Xie Y, Kang R, Klionsky DJ, Tang D. GPX4 in cell death, autophagy, and disease. Autophagy. 2023;19:2621–38. 37272058 10.1080/15548627.2023.2218764 PMC10472888

[7] Hangauer MJ, Viswanathan VS, Ryan MJ, Bole D, Eaton JK, Matov A, et al. Drug-tolerant persister cancer cells are vulnerable to GPX4 inhibition. Nature. 2017;551:247–50. 29088702 10.1038/nature24297 PMC5933935

[8] Lei G, Zhang Y, Koppula P, Liu X, Zhang J, Lin SH, et al. The role of ferroptosis in ionizing radiation-induced cell death and tumor suppression. Cell Res. 2020;30:146–62. 31949285 10.1038/s41422-019-0263-3 PMC7015061

[9] Tsoi J, Robert L, Paraiso K, Galvan C, Sheu KM, Lay J, et al. Multi-stage differentiation defines melanoma subtypes with differential vulnerability to drug-induced iron-dependent oxidative stress. Cancer Cell. 2018;33:890–904.e5. 29657129 10.1016/j.ccell.2018.03.017 PMC5953834

[10] Viswanathan VS, Ryan MJ, Dhruv HD, Gill S, Eichhoff OM, Seashore-Ludlow B, et al. Dependency of a therapy-resistant state of cancer cells on a lipid peroxidase pathway. Nature. 2017;547:453–7. 28678785 10.1038/nature23007 PMC5667900

[11] Ye LF, Chaudhary KR, Zandkarimi F, Harken AD, Kinslow CJ, Upadhyayula PS, et al. Radiation-induced lipid peroxidation triggers ferroptosis and synergizes with ferroptosis inducers. ACS Chem Biol. 2020;15:469–84. 31899616 10.1021/acschembio.9b00939 PMC7180072

[12] Zhang C, Liu X, Jin S, Chen Y, Guo R. Ferroptosis in cancer therapy: a novel approach to reversing drug resistance. Mol Cancer. 2022;21:47. 35151318 10.1186/s12943-022-01530-y PMC8840702

[13] Wynn TA, Vannella KM. Macrophages in tissue repair, regeneration, and fibrosis. Immunity. 2016;44:450–62. 26982353 10.1016/j.immuni.2016.02.015 PMC4794754

[14] Lin Y, Xu J, Lan H. Tumor-associated macrophages in tumor metastasis: biological roles and clinical therapeutic applications. J Hematol Oncol. 2019;12:76. 31300030 10.1186/s13045-019-0760-3 PMC6626377

[15] Geiger-Maor A, Levi I, Even-Ram S, Smith Y, Bowdish DM, Nussbaum G, et al. Cells exposed to sublethal oxidative stress selectively attract monocytes/macrophages via scavenger receptors and MyD88-mediated signaling. J Immunol. 2012;188:1234–44. 22219328 10.4049/jimmunol.1101740

[16] Geiger-Maor A, Guedj A, Even-Ram S, Smith Y, Galun E, Rachmilewitz J. Macrophages regulate the systemic response to DNA damage by a cell non-autonomous mechanism. Cancer Res. 2015;75:2663–73. 25977329 10.1158/0008-5472.CAN-14-3635

[17] Guedj A, Volman Y, Geiger-Maor A, Bolik J, Schumacher N, Kunzel S, et al. Gut microbiota shape ‘inflamm-ageing’ cytokines and account for age-dependent decline in DNA damage repair. Gut. 2020;69:1064–75. 31586932 10.1136/gutjnl-2019-318491

[18] Shi L, Liu Y, Li M, Luo Z. Emerging roles of ferroptosis in the tumor immune landscape: from danger signals to anti-tumor immunity. FEBS J. 2021;289:3655–65. 10.1111/febs.16034 34042258

[19] Yoshida M, Minagawa S, Araya J, Sakamoto T, Hara H, Tsubouchi K, et al. Involvement of cigarette smoke-induced epithelial cell ferroptosis in COPD pathogenesis. Nat Commun. 2019;10:3145. 31316058 10.1038/s41467-019-10991-7 PMC6637122

[20] Skouta R, Dixon SJ, Wang J, Dunn DE, Orman M, Shimada K, et al. Ferrostatins inhibit oxidative lipid damage and cell death in diverse disease models. J Am Chem Soc. 2014;136:4551–6. 24592866 10.1021/ja411006a PMC3985476

[21] Beharier O, Tyurin VA, Goff JP, Guerrero-Santoro J, Kajiwara K, Chu T, et al. PLA2G6 guards placental trophoblasts against ferroptotic injury. Proc Natl Acad Sci USA. 2020;117:27319–28. 33087576 10.1073/pnas.2009201117 PMC7959495

[22] Slee EA, Zhu H, Chow SC, MacFarlane M, Nicholson DW, Cohen GM. Benzyloxycarbonyl-Val-Ala-Asp (OMe) fluoromethylketone (Z-VAD.FMK) inhibits apoptosis by blocking the processing of CPP32. Biochem J. 1996;315:21–4. (Pt 1). 8670109 10.1042/bj3150021 PMC1217173

[23] Yamamoto A, Tagawa Y, Yoshimori T, Moriyama Y, Masaki R, Tashiro Y. Bafilomycin A1 prevents maturation of autophagic vacuoles by inhibiting fusion between autophagosomes and lysosomes in rat hepatoma cell line, H-4-II-E cells. Cell Struct Funct. 1998;23:33–42. 9639028 10.1247/csf.23.33

[24] Yang WS, Stockwell BR. Ferroptosis: death by lipid peroxidation. Trends Cell Biol. 2016;26:165–76. 26653790 10.1016/j.tcb.2015.10.014 PMC4764384

[25] Wiernicki B, Dubois H, Tyurina YY, Hassannia B, Bayir H, Kagan VE, et al. Excessive phospholipid peroxidation distinguishes ferroptosis from other cell death modes including pyroptosis. Cell Death Dis. 2020;11:922. 33110056 10.1038/s41419-020-03118-0 PMC7591475

[26] Kajiwara K, Beharier O, Chng CP, Goff JP, Ouyang Y, Croix CM. Ferroptosis induces membrane blebbing in placental trophoblasts. J. Cell Sci. 2022;9:e20507. 10.1242/jcs.255737 PMC8084576 33414166

[27] Kim R, Hashimoto A, Markosyan N, Tyurin VA, Tyurina YY, Kar G, et al. Ferroptosis of tumour neutrophils causes immune suppression in cancer. Nature. 2022;612:338–46. 36385526 10.1038/s41586-022-05443-0 PMC9875862

[28] Bi C, Fu Y, Zhang Z, Li B. Prostaglandin E2 confers protection against diabetic coronary atherosclerosis by stimulating M2 macrophage polarization via the activation of the CREB/BDNF/TrkB signaling pathway. FASEB J. 2020;34:7360–71. 32350920 10.1096/fj.201902055R

[29] Tseng D, Volkmer JP, Willingham SB, Contreras-Trujillo H, Fathman JW, Fernhoff NB, et al. Anti-CD47 antibody-mediated phagocytosis of cancer by macrophages primes an effective antitumor T-cell response. Proc Natl Acad Sci USA. 2013;110:11103–8. 23690610 10.1073/pnas.1305569110 PMC3703977

[30] Kelley SM, Ravichandran KS. Putting the brakes on phagocytosis: “don’t-eat-me” signaling in physiology and disease. EMBO Rep. 2021;22:e52564. 34041845 10.15252/embr.202152564 PMC8183410

[31] Zhao J, Chow RP, McLeese RH, Hookham MB, Lyons TJ, Yu JY. Modelling preeclampsia: a comparative analysis of the common human trophoblast cell lines. FASEB Bioadv. 2021;3:23–35. 33521587 10.1096/fba.2020-00057 PMC7805545

[32] Schoots MH, Bourgonje MF, Bourgonje AR, Prins JR, van Hoorn EGM, Abdulle AE, et al. Oxidative stress biomarkers in fetal growth restriction with and without preeclampsia. Placenta. 2021;115:87–96. 34583270 10.1016/j.placenta.2021.09.013

[33] Zou Y, Henry WS, Ricq EL, Graham ET, Phadnis VV, Maretich P, et al. Plasticity of ether lipids promotes ferroptosis susceptibility and evasion. Nature. 2020;585:603–8. 32939090 10.1038/s41586-020-2732-8 PMC8051864

[34] Mbah NE, Lyssiotis CA. Metabolic regulation of ferroptosis in the tumor microenvironment. J Biol Chem. 2022;298:101617. 35065965 10.1016/j.jbc.2022.101617 PMC8892088

[35] Kapralov AA, Yang Q, Dar HH, Tyurina YY, Anthonymuthu TS, Kim R, et al. Redox lipid reprogramming commands susceptibility of macrophages and microglia to ferroptotic death. Nat Chem Biol. 2020;16:278–90. 32080625 10.1038/s41589-019-0462-8 PMC7233108

[36] Konishi H, Haga Y, Okumura M, Tsujino H, Higashisaka K, Tsutsumi Y. Coculture with macrophages alters ferroptosis susceptibility of triple-negative cancer cells. Cell Death Discov. 2024;10:108. 38429255 10.1038/s41420-024-01884-w PMC10907599

[37] Bersuker K, Hendricks JM, Li Z, Magtanong L, Ford B, Tang PH, et al. The CoQ oxidoreductase FSP1 acts parallel to GPX4 to inhibit ferroptosis. Nature. 2019;575:688–92. 31634900 10.1038/s41586-019-1705-2 PMC6883167

[38] Doll S, Freitas FP, Shah R, Aldrovandi M, da Silva MC, Ingold I, et al. FSP1 is a glutathione-independent ferroptosis suppressor. Nature. 2019;575:693–8. 31634899 10.1038/s41586-019-1707-0

[39] Doll S, Proneth B, Tyurina YY, Panzilius E, Kobayashi S, Ingold I, et al. ACSL4 dictates ferroptosis sensitivity by shaping cellular lipid composition. Nat Chem Biol. 2017;13:91–8. 27842070 10.1038/nchembio.2239 PMC5610546

[40] Yang JS, Morris AJ, Kamizaki K, Chen J, Stark J, Oldham WM, et al. ALDH7A1 protects against ferroptosis by generating membrane NADH and regulating FSP1. Cell. 2025;188:2569–85.e20. 40233740 10.1016/j.cell.2025.03.019 PMC12085291

[41] Khalaji A, Yancheshmeh FB, Farham F, Khorram A, Sheshbolouki S, Zokaei M, et al. Don’t eat me/eat me signals as a novel strategy in cancer immunotherapy. Heliyon. 2023;9:e20507. 37822610 10.1016/j.heliyon.2023.e20507 PMC10562801

[42] Kloditz K, Fadeel B. Three cell deaths and a funeral: macrophage clearance of cells undergoing distinct modes of cell death. Cell Death Discov. 2019;5:65. 30774993 10.1038/s41420-019-0146-x PMC6368547

[43] Ghantous L, Volman Y, Hefez R, Wald O, Stern E, Friehmann T, et al. The DNA damage response pathway regulates the expression of the immune checkpoint CD47. Commun Biol. 2023;6:245. 36882648 10.1038/s42003-023-04615-6 PMC9992352

