# Supplementary material for: Macrophages rescue cells from ferroptotic death
Source: Cell Death Dis. 2025 Dec 1;17(1):66. doi: 10.1038/s41419-025-08277-6 (PMC12827969; doi:10.1038/s41419-025-08277-6)
Supplement: Supplementary file 1 — Supplementary data [file 41419_2025_8277_MOESM1_ESM.docx]

**Supplementary data: Macrophages rescue cells from ferroptotic death**

**Supplementary Figure 1:** *Flow cytometry gating strategy*

To distinguish macrophages from ferroptotic cells during flow cytometry, macrophages were pre-labeled with CellTrace™ Far Red prior to co-culture. For the analysis of ferroptotic cell survival, live cells were identified by their exclusion of propidium iodide (PI), while macrophages were excluded based on CellTrace fluorescence. The number of viable cancer cells was then quantified.

**Supplementary Figure 2:** *Macrophages rescue cells treated with various doses of RSL3 and with ML162*

**a.** The indicated cell types were treated with the various RSL3 doses for 1h and then were incubated for 24h with and without Far-red-labeled THP-1-derived macrophages. Cells survival was determined by flow cytometry after gating out macrophages. **b.** Cells were treated with ML162 for 1h and then were incubated for 24h with and without Far-red-labeled THP-1-derived macrophages. Cells survival was determined by flow cytometry after gating out macrophages. **c.** Cell death in b was determined by LDH release. Graphs show an average (± STD) of at least triplicates in each group. Representative of three independent experiments is shown. ns: non-significant; ** p<0.01; *** p<0.001; **** p < 0.0001.

**Supplementary Figure 3:** *RSL3 Preferentially Induces Ferroptosis*

Cells were treated with RSL3 (500nM) to induce ferroptosis. To confirm specificity, cells were co-treated with either ferrostatin-1 (Fer-1; 0.5µM), Z-VAD-FMK (20µM), or Baf-A1 (100nM). Cell viability was assessed by flow cytometry after 24h. Graphs show an average (± STD) of 6 replicates in each group. Representative of three independent experiments is shown. ns: non-significant; ** p<0.01; **** p < 0.0001.

**Supplementary Figure 4**: *Imaging of macrophages adhering to and rescuing cells undergoing ferroptosis.*

U87MG and A375 cells were either left untreated or treated with RSL3 for 1h and then washed. CellTrace-Red labeled macrophages were then added (designated as time 0), and the co-cultures were incubated for 24 hours. Live cell imaging was performed with IncuCyte.

**Supplementary Figure 5:** *Dose dependent rescue of ferrototic cells by macrophages*

Cells were treated as in figure 2a and then co-cultured with different numbers of THP-1-derived macrophages. Cells survival was determined by flow cytometry after gating out macrophages. Graphs show an average (± STD) of triplicates in each group. Representative of three independent experiments is shown. ns: non-significant; ** p<0.01; *** p<0.001; **** p < 0.0001.

**Supplementary Figure 6**: *Characterization of M0, M1 and M2 macrophages*

Representative flow cytometric histograms showing M0, M1 and M2 macrophages immune-stained with anti CD11c, CD163 and CD86.

**Supplementary Figure 7**: *CD47 expression in ferroptotic and apoptotic cells*

Representative histogram of CD47 staining and flow cytometric analysis before (control) and 2h and 5h after ferroptosis (RSL3) or apoptosis (Staurosporine) induction in BeWo, A375 and U87MG cells.

**Supplementary Movies 1-2:** Time-lapse movies generated by confocal microscope.

**Supplementary Movies 3-7:** Time-lapse movies generated by IncuCyte.
